# Central venous catheter–associated complications in pediatric patients diagnosed with Hodgkin lymphoma: implications for catheter choice

**DOI:** 10.1007/s00520-022-07256-3

**Published:** 2022-07-01

**Authors:** Ceder H. van den Bosch, Judith Spijkerman, Marc H. W. A. Wijnen, Idske C. L. Kremer Hovinga, Friederike A. G. Meyer-Wentrup, Alida F. W. van der Steeg, Marianne D. van de Wetering, Marta Fiocco, Indra E. Morsing, Auke Beishuizen

**Affiliations:** 1 Princess Máxima Center for Pediatric Oncology, Utrecht, The Netherlands; 2 Van Creveldkliniek University Medical Centre Utrecht, Thrombosis and Hemostasis, Benign Hematology, Utrecht, The Netherlands; 3 Mathematical Institute, Leiden, The Netherlands; 4 Leiden University Medical Center, Leiden, The Netherlands

**Keywords:** Hodgkin lymphoma, Central venous catheter, Pediatric, Complications, Thrombosis

## Abstract

**Purpose:**

The purpose of this study was to determine the most optimal central venous catheter (CVC) for pediatric patients with Hodgkin lymphoma (HL) in terms of complications.

**Methods:**

A retrospective study including patients diagnosed with HL from 2015 to 2021 at the Princess Máxima Center was performed. Patients were followed from CVC insertion until removal or 06–2021, whichever came first. The primary outcome was the CVC-related complication incidence rate (IR) per 1000 CVC-days. Furthermore, the incidence rate ratio (IRR) was calculated by comparing complication IRs between peripherally inserted central catheters (PICC) and totally implantable venous access ports (TIVAP). Additionally, risk factors for central venous thrombosis (CVT) were identified.

**Results:**

A total of 98 patients were included. The most frequently observed complications were local irritation/infections (18%; *IR* 0.93), malfunctions (15%; *IR* 0.88), and CVC-related CVTs (10%; *IR* 0.52). Single lumen PICCs were associated with a higher risk of complications (49% vs. 26%; *IRR* 5.12, *CI*95% 2.76–9.50), severe complications (19% vs. 7%; *IRR* 11.96, *CI*95% 2.68–53.42), and early removal (18% vs. 7%; *IRR* 9.96, *CI*95% 2.18–45.47). A single lumen PICC was identified as a risk factor for CVC-related CVT when compared to TIVAPs (12% vs. 7%, *IRR* 6.98, *CI*95% 1.45–33.57).

**Conclusion:**

The insertion of a TIVAP rather than a PICC should be recommended for pediatric patients with HL, especially in the presence of CVT-related risk factors. Future trials should evaluate the efficacy and safety of direct oral anticoagulants for the primary prevention of CVT in pediatric patients with a PICC and other CVT-related risk factors.

**Supplementary Information:**

The online version contains supplementary material available at 10.1007/s00520-022-07256-3.

## Introduction

The vast majority of pediatric patients diagnosed with Hodgkin lymphoma (HL) will receive a central venous catheter (CVC) at the start of their treatment. Multiple CVC types are available, but single lumen totally implantable central venous access ports (TIVAP) and peripherally inserted central venous catheters (PICC) are the most frequently used CVCs in this patient group. Insertion of a PICC is still considered favorable in patients with HL since insertion is possible without the need for general anesthesia (which is especially favorable in children with mediastinal masses causing airway problems) and is considered safe because of the relatively short treatment period and larger peripheral vessels of this, usually older, pediatric patient group [1].

In contrast to PICCs, TIVAPs can stay in situ for a longer period and give patients more freedom of movement. However, general anesthesia is needed for insertion, sedation for removal, percutaneous punctures to access the port, and a larger scar will remain visible after removal, whereas these disadvantages do not apply to PICCs. On the other hand, higher incidence rates of mechanical failure, CVC-related infections, and CVC-related central venous thrombosis (CVT) have been associated with PICCs when compared to other CVC-types in a variety of adult and pediatric patients (i.e., oncology, intensive care unit, total parenteral nutrition) [1–10]. However, the incidence of all CVC-related complications for patients with HL specifically has not been described previously.

Based on studies in adults and children, the risk of CVC-related CVTs has been described to be higher in patients with HL, compared to other oncology patients [3, 9]. Suggested risk factors that may contribute to this difference are tumor-associated inflammation and compression of veins typically occurring in the upper body, the older age of patients, and frequent high-dose corticosteroid treatment, which are all known risk factors for thrombosis [1, 3, 11–15]. Since pediatric patients with HL might be at a higher risk of CVC-related CVTs and since the risk of other CVC-related complications per CVC type for this patient group is currently unknown, it is of importance that the most optimal CVC for these children is identified. In this study, we have analyzed all CVC complications and their outcomes.

## Methods

### Study design and participants

A retrospective study including all consecutive patients diagnosed with HL, who received a CVC and who were treated in the Princess Máxima Center for pediatric oncology (Utrecht, The Netherlands) from January 2015 until March 2021, was performed. Patients were excluded if their CVC was inserted in any other hospital than the Princess Máxima Center, if they were older than 18 years at CVC insertion, or if they did not give their consent to use their data for scientific research (*n* = 24). Each patient was followed up from first CVC insertion until first CVC removal or June 2021, whichever came first. Patients were treated following the guidelines of the European Network-Pediatric Hodgkin’s Lymphoma Study Group (EuroNet-PHL: C1, C2, or LP1) in an outpatient setting. The medical ethics committee of the University Medical Center Utrecht (Utrecht, The Netherlands) waived the need for official approval by the medical ethics committee (File number: 21/723).

### Data collection and definitions

The primary outcome was the incidence rate (IR) per 1000 CVC-days for all observed CVC-related complications in total and per CVC-type. This aggregated outcome was chosen to give an overview of the overall risk of CVC-related complications for pediatric HL patients. Secondary outcomes were the IRs of each complication type as described below per CVC-type, of early removal due to complications per CVC-type and of CVC-related CVTs per risk factor.

The patient files were assessed retrospectively for the occurrence of the following CVC-related complications: intra-operative complications, central line–associated bloodstream infections (CLABSI), local irritation/infection, CVC-related CVT, malfunctions, and mechanical complications (dislocations, ruptures, and dislodgement). Intra-operative complications were defined as any abnormalities during or directly after CVC insertion (e.g., pneumothorax, arterial puncture, dislocation, bleeding, malfunction). CLABSIs were defined following the Centers for Disease Control and Prevention criteria [16]. Local irritation/infections were defined as a positive exit-site culture, erythema, purulent drainage, or tenderness within 2 cm of the CVC track. CVTs were scored if their presence was confirmed by the radiology department in the imaging report. Imaging was performed due to the presence of CVT-related symptoms or for routine tumor response evaluation. Malfunctions were defined as the inability to flush and/or aspirate requiring the need of thrombolysis or CVC removal. Mechanical complications were defined as the detachment of CVC components, dislocation of the CVC diagnosed by a chest radiograph or a visible cuff, and rupture of the CVC components causing leakage. All complications were thereafter scored following the Clavien-Dindo classification [17]. Severe complications were defined as a Clavien-Dindo classification of III or higher. The Clavien-Dindo definitions are described in Online Resource 1 (Online Resource 1).

Furthermore, to evaluate the presence of CVT-related risk factors, the following data were collected: age, sex, obesity [18], HL type and stage following the Ann-Arbor classification [19, 20], presence of a mediastinal mass (i.e., confirmed by radiologist), smoking, thrombosis or laboratory-confirmed thrombophilia in medical (family) history, use of hormonal contraceptives, compression of the veins in the trajectory of the CVC as confirmed by a radiologist at insertion (for the vena cava superior specifically < 50% or > 50% compression), preference to not insert the CVC under general anesthesia as evaluated retrospectively by two pediatric lymphoma specialists, pediatric intensive care unit admission from diagnosis until end of study period, prophylactic anticoagulant use, signs of infection during CVT diagnosis, > one insertion attempt, CVC type, CVC side and lumen size, CVC use for total parenteral nutrition, and CVC to vein ratio for PICCs specifically. Additionally, the following information was extracted from the patient files: diagnosis date, CVC insertion date, end of treatment date, complication date, CVC insertion method, CVC insertion vein, reason for CVC removal, complication treatment, and hospital/intensive care unit admission due to complications. For CVT events specifically, the severity, symptoms, and complications (e.g., pulmonary embolism, CLABSI, vena cava superior syndrome, and post-thrombotic syndrome scored following the modified Villalta score [21]) were collected. If data was not explicitly reported in the patient files, this was reported as missing data.

### CVC insertion and maintenance

The vast majority of patients diagnosed with Hodgkin will receive either a PICC or TIVAP at the start of their treatment. A non-tunneled CVC is only inserted for a short period in case of an emergency setting, positive blood cultures, or if the insertion of a tunneled CVC is not possible. If a PICC or TIVAP is to be inserted, it is determined by the expected treatment duration, lumen needed, presence of a tumor causing airway problems, and the wishes of the patient. In case of an expected treatment duration of more than 6 months, the insertion of a TIVAP is recommended. In patients with a tumor causing airway problems, the insertion of a PICC is preferred to avoid the need for general anesthesia or a TIVAP is inserted after steroid treatment. In all other cases, the (dis)advantages of both CVC types are discussed and the patient is thereafter free of choice. All CVCs were inserted by a specialized vascular access team or a pediatric oncology surgeon. All CVCs were inserted ultrasound-guided, only by exception CVCs were inserted percutaneously based on anatomical land marks. The insertion vein was chosen based on the availability and quality of the veins (i.e., adequate blood-flow through vein and CVC-to-vein ratio of < 45% [22] for PICCs assessed by ultrasound), with a preference for the right jugular vein for the non-PICC CVCs. CVC care was performed by or under supervision of specialized pediatric oncology nurses following international guidelines [23, 24]. The CVCs were flushed with NaCl 0.9% before use and locked with heparin 100 international units per milliliter after every use. The locks were replaced once every 8 weeks for the TIVAP and once every week for the other CVC types if the CVC was not used.

### Statistical analysis

Differences between patients with a SL PICC and TIVAP with respect to baseline characteristics were analyzed using a Fisher Exact or Wilcoxon rank sum test, depending on the variable. The IRs per 1000 CVC-days were calculated with the number of all CVC-related events observed and total CVC-days (i.e., sum of the days from insertion until the end of follow-up, during in- and outpatient settings). Additionally, the IRs per 1000 CVC treatment days were calculated with the number of CVC-related events during the treatment period and total CVC-treatment days (i.e., sum of days during in- and outpatient settings from insertion until the end of follow-up or the last day of treatment, whichever came first; in case of a recurrence during insertion of the primary CVC, the days from recurrence diagnosis until the end of follow-up or last day of treatment, whichever came first, were added up to the total sum). This last calculation was performed since the frequency of complications might be higher during the treatment period and some CVCs remained in situ without treatment due to clinical follow-up and delays in the surgical lists.

Incidence rate ratios (IRR) along with their 95% confidence intervals (CI) were computed (1) to compare the IRs per 1000 CVC-days and CVC-treatment days for all complications between single lumen PICCs and TIVAPs and (2) to compare the IRs per 1000 CVC-days for CVC-related CVT between patients with and without CVT-related risk factors. The second analysis was performed for two different settings: (1) including only single lumen PICCs and TIVAPs (most commonly inserted CVCs) and (2) excluding patients where CVC insertion under general anesthesia was not preferred due to disease severity resulting in the insertion of a PICC instead of a TIVAP (since these patients possibly have a high risk of CVT and TIVAP insertion is not possible [1]). The exact confidence limits for the IRRs were computed based on the polynomial algorithm for person time data [25, 26]. The mean CVC to vein ratio for patients with a CVT compared to patients without a CVT was compared using an independent *t*-test. IBM SPSS Statistics for Windows version 26.0 (IBM Corp, USA) was used to perform all statistical analyses.

## Results

### Patient and CVC characteristics

In total 98 patients were included with a median age at diagnosis of 15 years (6–17). Most patients (96%) were diagnosed with classic HL. Compression of the veins due to lymphoma in the CVC tract was observed in 18 (18%) patients. Two patients (2%) received anticoagulants before CVC insertion and kept using it during CVC insertion due to a non-CVC-related thrombosis and venous compression (prophylactic dose *n* = 1, therapeutic dose *n* = 1). Additionally, one more patient (1%) received anticoagulants during CVC insertion due to venous compression (prophylactic dose *n* = 1). General anesthesia at diagnosis was not preferred in 14 (14%) patients, resulting in prephase therapy with steroids. In only five (36%) of these patients a PICC instead of a TIVAP was eventually inserted since general anesthesia was still not preferred (Table 1). Baseline characteristics for patients receiving a TIVAP or SL PICC are described separately in Online Resource 2. Patients with a SL PICC differed from patients in the TIVAP group in terms of age at diagnosis, Ann-Arbor stage, and CVC-(treatment) days (Online Resource 2).

**Table 1:** Baseline characteristics

Patient characteristics (N=98)			CVC characteristics (N=98)		
Sex, N (%)	Male	50 (51.0)	Days from diagnosis until insertion, median (range)		11 (0-41)
	Female	48 (49.0)	CVC-days, median; sum (range)		143; 19 341 (0-717)
Age at diagnosis, median (range)		15 (6-17)	CVC-treatment days, median; sum (range)		118; 11 158 (0-308)
Hodgkin type, N (%)	Classic	94 (95.9)	CVC type, N (%)	TIVAP	31 (31.6)
	NLPHL	4 (4.1)		SL PICC	57 (58.2)
Ann-Arbor staging, N (%)	I	2 (2.0)		DL PICC	9 (9.2)
	II	43 (43.9)		Non-tunneled	1 (1.0)^d^
	III	27 (27.6)	Introduction method, N (%)	Ultrasound	96 (98.0)
	IV	26 (26.5)		Anatomic landmarks	1 (1.0)
EuroNet-PHL protocol, N (%)	C1	2 (2.0)		Missing	1 (1.0)
	C2	92 (93.9)	Lumen number, N (%)	Single	88 (89.8)
	LP1	4 (4.1)		Double	9 (9.2)
Mediastinal mass, N (%)	No	8 (8.2)		Triple	1 (1.0)
	Yes	90 (91.8)	Lumen diameter, N (%)	<6.5 Fr	65 (66.3)
Obesity at diagnosis^a^, N (%)	No	80 (81.6)		≥6.5 Fr	31 (31.6)
	Yes	18 (18.4)		Missing	2 (2.0)
Smoking, N (%)	No	60 (61.2)	Insertion vein, N (%)	Jugular	29 (29.6)
	Yes	3 (3.1)		Subclavian	2 (2.0)
	Passive	6 (6.1)		Brachial	39 (39.8)
	Missing	29 (29.6)		Cephalic	1 (1.0)
Oral anti-conceptive use, N (%)	No	82 (83.7)		Basilica	26 (26.5)
	Progesterone	4 (4.1)		Femoral	1 (1.0)
	Progesterone and estrogen	12 (12.3)	Insertion side, N (%)	Right	86 (87.8)
Thrombophilia, N (%)	No	2 (20)		Left	12 (12.2)
	Yes	3 (3.1)	Long-term anticoagulant use during CVC-insertion^e^, N (%)	No	95 (96.9)
	Not tested	93 (94.9)		Prophylactic	1 (1.0)
Thrombotic family history, N (%)	Negative	61 (62.2)		Therapeutic	2 (2.0)
	Positive	5 (5.1)	>1 Insertion attempt, N (%)	No	92 (93.9)
	Missing	32 (32.7)		Yes	2 (2.0)
Compression veins, N (%)	No	80 (81.6)		Missing	4 (4.1)
	Yes	18 (18.4)	TPN over CVC, N (%)	TPN^f^	4 (4.1)
VCS compression, N (%)	No	84 (85.7)		No TPN	94 (95.9)
	<50%	10 (10.2)	CVC to vein ratio for PICCs, median (range)		0.27 (0.15-0.33)
	>50%	4 (4.1)			
Thrombosis before insertion, N (%)	No	97 (99.0)			
	Yes	1 (1.0)			
Anticoagulant use in period before and at insertion, N (%)	No	96 (98.0)			
	Prophylactic	1 (1.0)			
	Therapeutic	1 (1.0)			
CVC insertion under general anesthesia not preferred^b^, N (%)	No	93 (94.9)			
	Yes	5 (5.1)			
PICU admission, N (%)	No	95 (96.9)			
	Yes	3^c^ (3.1)			

*CVCs* central venous catheter, *NLPHL* nodular lymphocyte-predominant Hodgkin lymphoma, *PHL* pediatric Hodgkin lymphoma, *TIVAP* totally implantable venous access port, *PICC* peripherally inserted central catheter, *Fr* French, *VCS* vena cava superior, *SL* single lumen, *DL* double lumen, *TPN* total parenteral nutrition, *PICU* pediatric intensive care unit, *N* number.

^a^Obesity was scored following: Cole 2000^18^.

^b^Based on clinical evaluation by two lymphoma specialists.

^c^PICU admissions due to respiratory or circulatory insufficiency, CVT-related PICU admission registered as “no.”

^d^Inserted during an emergency setting due to an anaphylactic reaction to contrast.

^e^CVCs where thrombolytics were given for only a short period of time due to for example hospitalization or where thrombolytics were given after a CVT was observed are registered as “no.” Reasons for anticoagulant use were the following: not CVC-related thrombosis (*n* = 2) and venous compression (*n* = 1).

^f^Median (range) days of TPN: 4.5 (1–13)

Mainly single lumen CVCs (90%) were inserted; single lumen PICCs (65%) and TIVAPs (35%). The CVCs were in situ for a total of 19,341 CVC-days and 11,158 CVC-treatment days. TIVAPs were in situ for a median of 377 (33–717) days and single lumen PICCs for 105 (0–208) days. Of all CVCs, 12 (12%) were removed due to complications, 75 (77%) due to end of treatment, three (3%) due to the need for another CVC type, one (1%) since the patient was not content with the location, and seven (7%) were still in situ at the end of this study (Table 1).

### Complications

A total of 58 complications were observed with an IR of 3.00 per 1000 CVC-days. In 42% of all CVCs, at least one complication was observed. The most frequently observed complications per 1000 CVC-days were local irritation/infections (18%; *IR* 0.93), malfunctions (14%; *IR* 0.88), and CVC-related CVT (10%; *IR* 0.52). The IRs per 1000 CVC-treatment days were comparable or higher for all complication types. All complications were observed after a median of 63 (3–378) days. CVC-related CVT was the most frequently observed reason for early CVC removal (50% of CVCs removed early due to complications, 6% of all inserted CVCs). Hospital admission was mainly observed in patients experiencing a CLABSI. One patient was admitted to the intensive care unit due to a combined CVT and CLABSI episode (Table 2).

**Table 2:** Incidence of CVC-related complications

Complications	Events, *n* (%)	CVCs^e^, *n* (% all CVCs)	IR per 1000 CVC-days	IR per 1000 CVC-treatment days	Days until complication, median (range)	CVC removal, *n* (% all CVCs)	Hospital admission days, median (range)
Intra-operative	6 (10.3)	6 (6.1)	NA	NA	NA	1 (1.0)	0 (0–1)
CLABSI	3 (5.2)	3 (3.1)	0.16	0.27	19 (13–99)	1^a^ (1.0)	6 (5–10)
Local irritation/infection	18 (31.0)	18 (18.4)	0.93	1.43	66 (30–139)	2 (2.0)	0 (0–5)
CVT	10 (17.2)	10 (10.2)	0.52	0.81	19 (3–374)	6^a^ (6.1)	0 (0–28)
Dislocation	2 (3.4)	2 (2.0)	0.10	0.09	4 (3–5)	0 (0.0)	0 (0–0)
Malfunction	17^b^ (29.3)	14 (14.3)	0.88	1.43	67 (10–378)	2 (2.0)	0 (0–0)
Rupture	1 (1.4)	1 (1.0)	0.05	0.09	16 (16–16)	1 (1.0)	0 (0–0)
Dislodgement	1 (1.4)	1 (1.0)	0.05	0.09	39 (39–39)	0 (0.0)	0 (0–0)
Total	58 (100.0)	41 (41.8)^c^	3.00	4.75	63 (3–378)	12^d^ (12.2)	0 (0–28)

*CVCs* central venous catheter, *CLABSI* central line associated bloodstream infection, *CVT* central venous thrombosis, *IR* incidence rate. In total, five (8.6%) CVC-related complications were observed after the end of treatment. ^a^One CVC was removed due to a combined CVT and CLABSI episode. ^b^Malfunctions successfully treated with thrombolysis (*n* = 12), unsuccessful thrombolysis resulting in removal (*n* = 2), unsuccessful thrombolysis due to the presence of a CVT for which the CVC was removed (*n* = 1), unsuccessful thrombolysis after which malfunction was treated with pulsatile flushes of regular saline (*n* = 1). ^c^If multiple complications were identified in one CVC, this was counted as one to calculate this percentage. This means that 42.3% of all patients experienced one or more complications during CVC insertion. ^d^Combined CVT and CLABSI episode counted as one in total. ^e^For each complication type, only the first was counted per CVC

In total, ten CVC-related CVT events were observed, among which eight CVTs were identified due to symptoms and two due to a routine ultrasound and magnetic-resonance imaging for tumor response evaluation. In four patients, the CVT resulted in complications; three short-term complications (i.e., vena cava superior syndrome causing chylothorax, pulmonary embolisms, and septic thrombophlebitis); and one long-term complication (i.e., post-thrombotic syndrome; modified Villalta score 4 indicating a moderate post-thrombotic syndrome). The CVTs were diagnosed after a median of 19 (3–374) days after insertion. All CVT events were treated with anticoagulants; in six cases, the CVC was removed; one patient required a thrombectomy and another required a percutaneous transluminal angioplasty. None of the patients with a CVT received thrombosis prophylaxis before the CVT occurred. In four CVT cases, simultaneous clinical signs of infection were present (Online Resource 3).

### Complications TIVAP versus single lumen PICC

During CVC-insertion, single lumen PICCs were associated with a significantly higher risk of complications (49% vs. 26%; *IRR* 5.12, *CI*95%2.76–9.50) and removal due to complications (18% vs. 7%; *IRR* 9.96, *CI*95%2.18–45.47), when compared to TIVAPs during complete CVC insertion. Specifically, a higher risk of local irritation/infections (26% vs. 7%; *IRR* 14.95, *CI*95%3.42–65.35) and CVC-related CVTs (12% vs. 7%; *IRR* 6.98, *CI*95%1.45–33.57) was associated with the insertion of single lumen PICCs compared to TIVAPs. A Clavien-Dindo grade of I or II was scored for the vast majority of complications. Of all complications, a grade of III or higher was scored in 27.6%. Single lumen PICCs were associated with a significantly higher risk of severe complications, i.e., Clavien-Dindo grade of III or higher, when compared to TIVAPs (19% vs. 7%; *IRR* 11.96, *CI*95%2.68–53.42) (Table 3 and Online Resource 1).

**Table 3:** Comparison of CVC-related complication incidence rates between TIVAPs and single lumen PICCs

	TIVAP *n* = 31 12,258 CVC days and 4193 CVC-treatment days				Single lumen PICC *n* = 57 6151 CVC days and 6033 CVC-treatment days				IRR (*CI*95%)	
Complications	Events, *n* (%)	CVCs, *n* (% all CVCs)	IR per 1000 CVC days	IR per 1000 CVC treatment days	Events, *n* (%)	CVCs, *n* (% all CVCs)	IR per 1000 CVC days	IR per 1000 CVC treatment days	During CVC insertion	During treatment
Intra-operative	2 (14.3)	2 (6.5)	NA	NA	2 (5.6)	2 (3.5)	NA	NA	NA	NA
CLABSI	1 (7.1)	1 (3.2)	0.08	0.24	2 (5.6)	2 (3.5)	0.33	0.33	3.99 (0.36–43.95)	1.39 (0.13–15.33)
Local infection	2 (14.3)	2 (6.5)	0.16	0.48	15 (41.7)	15 (26.3)	2.44	2.15	14.95 (3.42–65.35)	4.52 (1.02–20.02)
CVT	2 (14.3)	2 (6.5)	0.16	0.24	7 (19.24)	7 (12.3)	1.14	1.16	6.98 (1.45–33.57)	4.87 (0.60–39.54)
Malfunction	6 (46.9)	4 (12.9)	0.49	1.19	8 (22.2)	6 (10.5)	1.30	1.33	2.66 (0.92–7.66)	1.11 (0.36–3.40)
Dislocation	0 (0.0)	0 (0.0)	0.00	0.00	1 (2.8)	1 (1.8)	0.16	0.17	Undefined	Undefined
Rupture	0 (0.0)	0 (0.0)	0.00	0.00	1 (2.8)	1 (1.8)	0.16	0.17	Undefined	Undefined
Dislodgement	1 (7.1)	1 (32)	0.08	0.24	0 (0.0)	0 (0.0)	0.00	0.00	Undefined	Undefined
Total	14 (100.0)	8 (25.8)	1.14	2.86	36 (100.0)	28 (49.1)	5.85	5.64	5.12 (2.76–9.50)	1.97 (1.02–3.80)
Clavien-Dindo grade of ≥ 3	2 (14.3)	2^a^ (6.5)	0.16	0.24	12 (33.3)	11^a^ (19.3)	1.95	1.99	11.96 (2.68–53.42)	8.34 (1.09–64.13)
Removal due to complications	2 (NA)	2 (6.5)	0.16	0.48	10 (NA)	10 (17.5)	1.63	1.66	9.96 (2.18–45.47)	3.48 (0.76–15.86)

*CVCs* central venous catheter, *CLABSI* central line associated bloodstream infection, *CVT* central venous thrombosis, *TIVAP* totally implantable venous access port, *PICC* peripherally inserted central catheter, *IR* incidence rate, *IRR* incidence rate ratio, *CI* confidence interval, *N* number, *NA* not applicable. The events, CVCs, and IR per 1000 CVC days columns include all CVC-related complications observed. The IR per 1000 CVC treatment days includes all CVC-related complications observed from CVC insertion until the end of treatment or removal of the first CVC, whichever comes first. ^a^Highest Clavien-Dindo grade per CVC counted

During treatment, single lumen PICCs were also associated with a significantly higher risk of complications (*IRR* 1.97, *CI*95% 1.02–3.80), local irritation/infections in particular (*IRR* 4.52, *CI*95% 1.02–20.02), and complications with a Clavien-Dindo grade of III or higher (*IRR* 8.34, *CI*95% 1.09–64.13) (Table 3 and Online Resource 1).

### Risk factors for CVC-related CVT

**Fig. 1:**
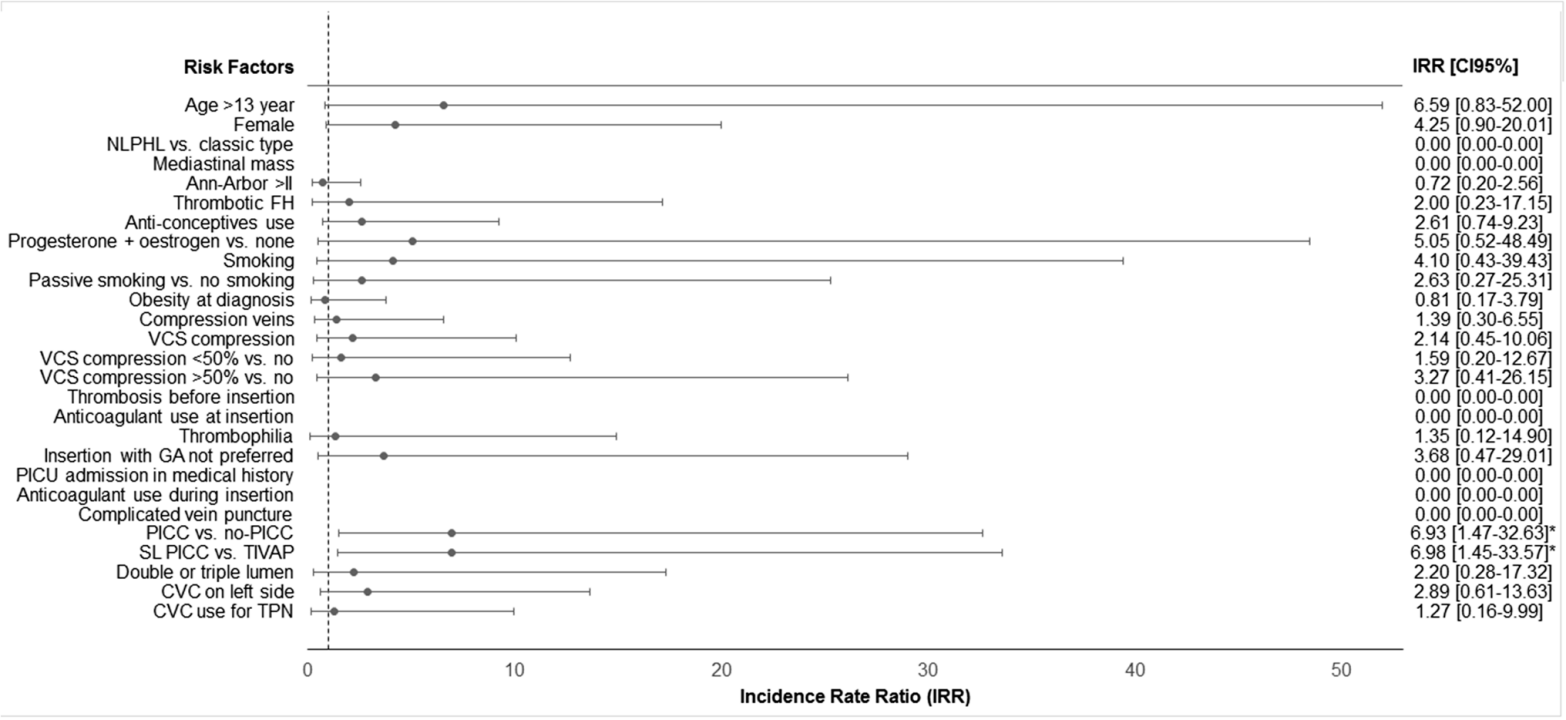
Risk factors for CVC-related CVT in pediatric Hodgkin lymphoma patients. CVC: central venous catheters, CVT: central venous thrombosis, TIVAP: totally implantable venous access port, PICC: peripherally inserted central catheter, FH: family history, NLPHL: nodular lymphocyte-predominant Hodgkin lymphoma, TPN: total parenteral nutrition, PICU: pediatric intensive care unit, SL: single lumen, GA: general anesthesia, VCS: vena cava superior, IRR: incidence rate ratio, CI: confidence interval. Mean CVC to vein ratio (calculated only for patients with a PICC) did not differ between patients with and without a CVC-related CVT (0.25 versus 0.27; *CI*95% − 0.02–0.06)

A (single lumen) PICC compared to a TIVAP or other non-PICC CVC was identified as the only significant risk factors for a CVC-related CVT. All other IRRs per CVT-related risk factor and their associated 95%*CI* are described in Fig. 1 and in more detail in the supporting information (Fig. 1 and Online Resource 4).

When the analysis was repeated twice including only single lumen PICCs and TIVAPs (*n* = 88) and excluding patients where general anesthesia was not preferred resulting in the insertion of a PICC instead of a TIVAP (*n* = 5), the insertion of a PICC was still identified as a risk factor. For patients with a single lumen PICC or TIVAP, only the female sex was additionally identified as a risk factor (Online Resource 5 and 6).

## Discussion

In this study, we investigated the incidence of CVC-related complications in patients treated for HL. We found at least one complication in 42% of all patients. Complications were more often observed, more severe, and resulted in more frequent early CVC removal in patients receiving a PICC compared to patients with a TIVAP. One of the most frequent and severe complications was a CVC-related CVT, which occurred in one out of ten patients with HL. The incidence rate of CVC-related CVT in this study was seven times higher for patients with a single lumen PICC compared to patients with a TIVAP.

CVT is a severe complication, as most patients will receive anticoagulant therapy for months and CVC replacement is often necessary. In severe cases, CVT-infections, vena cava superior syndrome, embolisms, and long-term complications like post-thrombotic syndrome can occur. In this study, a CVC-related CVT incidence of 10% was observed. This incidence falls within the wide range reported for children with cancer in general of 2–50% [27–29]. The risk of CVC-related CVTs has been described to be higher in patients with HL compared to other oncology patients, presumably caused by the frequent presence of risk factors for CVT (e.g., vein compression in the upper body, high-dose corticosteroid treatment), as also described in the “Introduction” section [1, 3, 9, 11–14, 28]. However, the difference between HL and non-HL patients might also be explained by the CVC types included in the previously performed studies, i.e., only 0–3% PICCs [28, 29]. Previous studies including lymphoma patients only reported CVC-related CVT incidences of 7–9% for adults [30] and 3–7% for pediatric patients [1, 14]. The low incidence of 3% (definite CVC-related CVT) as reported by Schonning et al. [14] might be explained by the inclusion of mainly patients with a TIVAP and that the other not-definite CVTs reported by the authors might also have been related to the CVC.

The results of this study suggest that single lumen PICCs are associated with much higher complication rates (49% vs. 26%; *IRR* 5.12), early removal (18% vs. 7%; *IRR* 9.96), and more severe complications (19% vs. 7%; *IRR* 11.96) when compared to TIVAPs. The high rate of complications associated with PICCs has also been previously reported in a variety of patient populations [1–9]. In this study, the incidence rate of local irritation/infections and CVC-related CVTs specifically was higher in patients receiving single lumen PICCs compared to TIVAPs (26% vs. 7%; *IRR* 14.95 and 12% vs. 7%; *IRR* 6.98, respectively), suggesting that TIVAPs are more suitable for this patient group compared to PICCs in terms of CVC-related complications and the risk of early removal. However, due to the non-randomized retrospective nature of this study, differences in baseline characteristics between patients with a SL PICC and TIVAP were observed. Patients with a SL PICC were slightly older, were more often diagnosed with Ann-Arbor stage I or II, and had their CVC in situ for a shorter (treatment) period compared to patients with a TIVAP. The older age might be explained by the fact that PICC insertion without anesthesia is less preferable in younger patients. This might result in some bias since older age has been described to be associated with a higher incidence of CVT [1]. Furthermore, patients with a higher Ann-Arbor stage are more frequently expected to have a longer treatment duration, resulting in the more frequent insertion of a TIVAP in this group. Higher rates of CVT in children and adults with higher Ann-arbor stage have been previously described, but these results were not significant [11, 15]. In this study however, patients with a TIVAP developed less CVTs compared to patients with a PICC. The lower number of CVC-(treatment) days might be explained by the fact that PICCs are removed much sooner after the end of treatment. This can be explained by two reasons: (1) TIVAPs are more often left in situ for the clinical follow-up period compared to PICCs and (2) the surgical waiting lists for TIVAP removal. Since the risk of some complications might be higher during treatment (i.e., intensive use of CVC), the IRs per 1000 CVC-treatment days were also calculated, which still showed that the insertion of a SL PICC is associated with a significantly higher risk of (severe) complications and local infections in particular compared to a TIVAP.

The high rate of complications associated with PICCs was also reported previously in adult patients with HL and pediatric oncology patients in general [1, 9–13]. Three studies did investigate risk factors for CVT (CVC and non-CVC-related) specifically in pediatric patients with lymphoma. Gartrell et al. [1] identified the insertion of a PICC as a risk factor, but noted that this result might be biased since patients unstable for sedation with large mediastinal masses initially received a PICC. Insertion of a PICC as an independent risk factor for CVC-related CVT was not observed by Athale et al. [15] and Schonning et al. [14]; however, almost all patients included in these studies received a TIVAP. Furthermore, Athale et al. [15] identified the presence of a mediastinal mass as a risk factor. Schonning et al. [14] did not identify any risk factors [1, 14, 15]. Previous studies suggested that the device-specific quality of life was lower and costs were higher for oncology patients in general with a PICC compared to a TIVAP [4, 31]. Taxbro et al. [31] pointed out that this increase in costs was mainly caused by the costs related to complications.

Based on the complications found in this study together with the current literature, the insertion of a TIVAP rather than a PICC should be advised by physicians in pediatric patients diagnosed with HL, especially in case of CVT-related risk factors. This is in line with the conclusions drawn by Taxbro et al. [6] for oncology patients in general.

If a PICC is preferred, for example since general anesthesia is preferably avoided, the use of prophylactic anticoagulants could be considered, particularly when other risk factors for thrombosis in pediatric oncology patients like age, sex, thrombophilia, and vein compression are present [1]. In adult oncology patients with a PICC, the use of direct oral anticoagulants (DOAC) and low-molecular weight heparin for primary CVT prevention resulted in a significant decrease in the incidence of CVT with comparable safety outcomes [32, 33], but the evidence to use primary prophylaxis for patients with cancer and a CVC is still scarce and guidelines therefore do not recommend primary prophylaxis [34]. The first results of phase III trials investigating DOACs in children showed that DOACs are at least as efficient and safe as low-molecular weight heparin and vitamin K antagonists for the treatment and secondary prophylaxis of thrombotic events in children with different clinical conditions [35–38]. Future trials should be focusing on the use of DOACs as primary prevention for pediatric patients.

This study shows the high risk of CVC-related complications associated with pediatric patients diagnosed with HL receiving a PICC. Strengths are that this study describes a large pediatric HL cohort, that this study investigates all CVC-related complications, that CVC-related complication severity and outcomes were investigated, that multiple CVT-related risk factors were evaluated, and that separate analyses were performed excluding patients requiring a PICC instead of a TIVAP since CVC insertion under general anesthesia was not preferred. Limitations of this study are the retrospective study design, differences in baseline characteristics between the SL PICC and TIVAP group, and the impossibility to perform a multivariate analysis due to the small patient group.

In conclusion, PICCs were associated with a higher risk of (severe) complications, CVTs specifically, and subsequent CVC removal when compared to TIVAPs. The insertion of a TIVAP rather than a PICC should therefore be advised by physicians in pediatric patients diagnosed with HL, especially in case of CVT-related risk factors. Future trials should evaluate the efficacy and safety of direct oral anticoagulants for the primary prevention of CVT in pediatric patients with a PICC and other CVT-related risk factors.

## Supplementary Information

Below is the link to the electronic supplementary material.

Supplementary file1 (DOCX 18 KB)

Supplementary file2 (DOCX 18 KB)

Supplementary file3 (DOCX 19 KB)

Supplementary file4 (DOCX 26 KB)

Supplementary file5 (DOCX 21 KB)

Supplementary file6 (DOCX 20 KB)

## Data Availability

The data that support the findings of this study are available on request from the corresponding author. The data are not publicly available due to privacy or ethical restrictions.

## Code availability

The custom code that supports the findings of this study is available on request from the corresponding author.
