# Supplementary material for: Central venous catheter–associated complications in pediatric patients diagnosed with Hodgkin lymphoma: implications for catheter choice
Source: Support Care Cancer. 2022 Jul 1;30(10):8069–79. doi: 10.1007/s00520-022-07256-3 (PMC9512752; doi:10.1007/s00520-022-07256-3)
Supplement: Supplementary file 1 — Supplementary file1 (DOCX 18 KB) [file 520_2022_7256_MOESM1_ESM.docx]

Central venous catheter associated complications in pediatric patients diagnosed with Hodgkin lymphoma: implications for catheter choice

**Journal title:** Supportive Care in Cancer

**Authors:** Ceder H. van den Bosch^1^, Judith Spijkerman^1^, Marc H.W.A. Wijnen^1^, Idske C.L. Kremer Hovinga^2^, Friederike A.G. Meyer-Wentrup ^1^, Alida F.W. van der Steeg^1^, Marianne D. van de Wetering^1^, Marta Fiocco^1,3,4^, Indra E. Morsing^1^, Auke Beishuizen^1^.

**Author affiliations:**

^1^ Princess Máxima Center for Pediatric Oncology, Utrecht, The Netherlands.

^2^ Van Creveldkliniek University Medical Centre Utrecht, Benign Hematology, Thrombosis and Hemostasis, Utrecht, The Netherlands.

^3^ Mathematical Institute, Leiden, The Netherlands

^4^ Leiden University Medical Center, Leiden, The Netherlands.

**Details corresponding author:**

C.H. van den Bosch, M.D. / PhD-student

C.H.vandenBosch-4@prinsesmaximacentrum.nl

ORCHID ID: 0000-0003-0612-578X

ONLINE RESOURCE 1 Clavien-Dindo classification

| Grade | Definition | Complications total, n (%) | Complications TIVAPs, n (%) | Complications SL PICCs, n (%) |
| --- | --- | --- | --- | --- |
| Grade I | Any deviation from the normal postoperative course without the need for pharmacological treatment or surgical, endoscopic and radiological interventions^a^ | 22 (37.9) | 5 (35.7) | 14 (38.9) |
| Grade II | Requiring pharmacological treatment with drugs other than such allowed for grade I complications. | 20 (34.5) | 7 (50.0) | 10 (27.8) |
| Grade III | Requiring surgical, endoscopic or radiological intervention | 15 (25.9) | 2 (14.3) | 11 (30.6) |
| IIIa | Intervention not under general anesthesia | 12 (20.7) | 0 (0.0) | 10 (27.8) |
| IIIb | Intervention under general anesthesia | 3 (5.2) | 2 (14.3) | 1 (2.8) |
| Grade IV | Life-threatening complication (including CNS complications)* requiring ICU-management | 1 (1.7) | 0 (0.0) | 1 (2.8) |
| IVa | Single organ dysfunction | 1 (1.7) | 0 (0.0) | 1 (2.8) |
| IVb | Multi organ dysfunction | 0 (0.0) | 0 (0.0) | 0 (0.0) |
| Grade V | Death | 0 (0.0) | 0 (0.0) | 0 (0.0) |
| Total |  | 58 (100.0) | 14 (100.0) | 36 (100.0) |

TIVAP; Totally Implantable Venous Access Port, PICC; Peripherally Inserted Central Catheter, SL; Single Lumen, CNS; Central Nervous System, ICU; Intensive Care Unit.

^a^Allowed therapeutic regimens are: drugs as antiemetics, antipyretics, analgesics, diuretics and electrolytes and physiotherapy.
