# Supplementary material for: Central venous catheter–associated complications in pediatric patients diagnosed with Hodgkin lymphoma: implications for catheter choice
Source: Support Care Cancer. 2022 Jul 1;30(10):8069–79. doi: 10.1007/s00520-022-07256-3 (PMC9512752; doi:10.1007/s00520-022-07256-3)
Supplement: Supplementary file 2 — Supplementary file2 (DOCX 18 KB) [file 520_2022_7256_MOESM2_ESM.docx]

Central venous catheter associated complications in pediatric patients diagnosed with Hodgkin lymphoma: implications for catheter choice

**Journal title:** Supportive Care in Cancer

**Authors:** Ceder H. van den Bosch^1^, Judith Spijkerman^1^, Marc H.W.A. Wijnen^1^, Idske C.L. Kremer Hovinga^2^, Friederike A.G. Meyer-Wentrup ^1^, Alida F.W. van der Steeg^1^, Marianne D. van de Wetering^1^, Marta Fiocco^1,3,4^, Indra E. Morsing^1^, Auke Beishuizen^1^.

**Author affiliations:**

^1^ Princess Máxima Center for Pediatric Oncology, Utrecht, The Netherlands.

^2^ Van Creveldkliniek University Medical Centre Utrecht, Benign Hematology, Thrombosis and Hemostasis, Utrecht, The Netherlands.

^3^ Mathematical Institute, Leiden, The Netherlands

^4^ Leiden University Medical Center, Leiden, The Netherlands.

**Details corresponding author:**

C.H. van den Bosch, M.D. / PhD-student

C.H.vandenBosch-4@prinsesmaximacentrum.nl

ORCHID ID: 0000-0003-0612-578X

ONLINE RESOURCE 2 Baseline characteristics for TIVAP and SL PICC group

|  |  | **TIVAP**  **N=31** | **SL PICC**  **N=57** | **p-value^e^** |
| --- | --- | --- | --- | --- |
| **Sex, N (%)** | Male | 18 (58.1) | 28 (49.1) | 1.00 |
|  | Female | 13 (41.9) | 29 (50.9) |  |
| **Age at diagnosis, median (range)** |  | 13 (6-17) | 15 (8-17) | 0.02* |
| **Hodgkin type, N (%)** | Classic | 31 (100.0) | 53 (93.0) | 0.29 |
|  | NLPHL | 0 (0.0) | 4 (7.0) |  |
| **Ann-Arbor staging, N (%)** | I | 0 (0.0) | 2 (3.5) | 0.01* |
|  | II | 7 (22.6) | 31 (54.4) |  |
|  | III | 12 (38.7) | 14 (24.6) |  |
|  | IV | 12 (38.7) | 10 (17.5) |  |
| **EuroNet-PHL protocol C1/C2, N (%)** | C1 | 2 (6.5) | 0 (0.0) | 0.13 |
|  | C2 | 29 (93.5) | 53 (93.0) |  |
| **Mediastinal mass, N (%)** | No | 2 (6.5) | 6 (10.5) | 0.71 |
|  | Yes | 29 (93.5) | 51 (89.5) |  |
| **Obesity at diagnosis^a^, N (%)** | No | 25 (80.6) | 49 (86.0) |  |
|  | Yes | 6 (19.4) | 8 (14.0) |  |
| **Smoking, N (%)** | No | 22 (71.0) | 33 (57.9) | 0.22 |
|  | Yes | 2 (6.5) | 1 (1.8) |  |
|  | Passive | 4 (12.9) | 1 (1.8) |  |
|  | Missing | 3 (9.7) | 22 (38.6) |  |
| **Oral anti-conceptive use , N (%)** | No | 24 (77.4) | 49 (86.0) | 0.28 |
|  | Progesterone | 3 (9.7) | 1 (1.8) |  |
|  | Progesterone and estrogen | 4 (12.9) | 7 (12.3) |  |
| **Thrombophilia, N (%)** | No | 0 (0.0) | 1 (1.8) | 0.71 |
|  | Yes | 0 (0.0) | 3 (5.3) |  |
|  | Not tested | 31 (100.0) | 53 (93.0) |  |
| **Thrombotic family history, N (%)** | Negative | 17 (54.8) | 36 (63.2) | 0.59 |
|  | Positive | 2 (6.5) | 2 (3.5) |  |
|  | Missing | 12 (38.7) | 19 (33.3) |  |
| **Compression veins, N (%)** | No | 29 (93.5) | 44 (77.2) | 0.07 |
|  | Yes | 2 (6.5) | 13 (22.8) |  |
| **VCS compression, N (%)** | No | 30 (96.8) | 46 (80.7) | 0.12 |
|  | <50% | 1 (3.2) | 7 (12.3) |  |
|  | >50% | 0 (0.0) | 4 (7.0) |  |
| **Thrombosis before insertion, N (%)** | No | 31 (100.0) | 56 (98.2) | 1.00 |
|  | Yes | 0 (0.0) | 1 (1.8) |  |
| **Anticoagulant use in period before and at insertion, N (%)** | No | 31 (100.0) | 54 (94.7) | 0.54 |
|  | Prophylactic | 0 (0.0) | 1 (1.8) |  |
|  | Therapeutic | 0 (0.0) | 1 (1.8) |  |
| **CVC insertion under general anesthesia not preferred^b^, N (%)** | No | 31 (100.0) | 52 (91.2) | 0.16 |
|  | Yes | 0 (0.0) | 5 (8.8) |  |
| **PICU admission^c^, N (%)** | No | 31 (100.0) | 55 (96.5) | 0.54 |
|  | Yes | 0 (0.0) | 2 (3.5) |  |
| **Days from diagnosis until insertion, median (range)** |  | 14 (0-41) | 11 (0-36) | 0.06 |
| **CVC-days, median; sum (range)** |  | 377; 12.258 (33-717) | 105; 6.151 (0-208) | 0.00* |
| **CVC-treatment days, median; sum (range)** |  | 130; 4 193 (33-308) | 105; 6 033 (0-208) | 0.04* |
| **Introduction method, N (%)** | Ultrasound | 31 (100.0) | 56 (98.2) | 1.00 |
|  | Anatomic landmarks | 0 (0.0) | 1 (1.8) |  |
| **Lumen diameter, N (%)** | <6.5 Fr | 0 (0.0 | 56 (98.2) | NA |
|  | ≥6.5 Fr | 31 (100.0) | 0 (0.0) |  |
|  | Missing | 0 (0.0 | 1 (1.8) |  |
| **Insertion vein, N (%)** | Jugular | 29 (93.5) | 0 (0.0) | NA |
|  | Subclavian | 2 (6.5) | 0 (0.0) |  |
|  | Brachial | 0 (0.0) | 33 (57.9) |  |
|  | Cephalic | 0 (0.0) | 1 (1.8) |  |
|  | Basilica | 0 (0.0) | 23 (40.4) |  |
| **Insertion side, N (%)** | Right | 29 (93.5) | 48 (84.2) | 0.32 |
|  | Left | 2 (6.5) | 9 (15.8) |  |
| **Long-term anticoagulant use during CVC-insertion^d^, N (%)** | No | 31 (100.0) | 54 (94.7) | 0.70 |
|  | Prophylactic | 0 (0.0) | 1 (1.8) |  |
|  | Therapeutic | 0 (0.0) | 2 (3.5) |  |
| **>1 Insertion attempt, N (%)** | No | 30 (96.8) | 53 (93.0) | 0.79 |
|  | Yes | 0 (0.0) | 2 (3.5) |  |
|  | Missing | 1 (3.2) | 2 (3.5) |  |
| **TPN over CVC, N (%)** | TPN^f^ | 3 (9.7) | 1 (1.8) | 0.12 |
|  | No TPN | 28 (90.3) | 56 (98.2) |  |
| **CVC to vein ratio for PICCs, median (range)** |  |  | 0.27 (0.15-0.33) | NA |

CVCs; Central Venous Catheter, NLPHL; Nodular lymphocyte-predominant Hodgkin lymphoma, PHL; Pediatric Hodgkin Lymphoma, TIVAP; Totally Implantable Venous Access Port, PICC; Peripherally Inserted Central Catheter, Fr; French, VCS; Vena Cava Superior, SL; Single lumen, TPN; Total Parenteral Nutrition; PICU; Pediatric Intensive Care Unit; N; Number, NA; Not applicable.

^a^ Obesity was scored following: Cole 2000^18^

^b^ Based on clinical evaluation by two lymphoma specialists.

^c^ PICU admissions due to respiratory or circulatory insufficiency, CVT-related PICU admission registered as “No”.

^d^ CVCs where thrombolytics were given for only a short period of time due to for example hospitalization or where thrombolytics were given after a CVT was observed are registered as “No”. Reasons for anticoagulant use were: not-CVC related thrombosis (n=2) and venous compression (n=1).

^e^ Fisher exact or Wilcoxon rank sum test, depending on the variable;

*Significant values
