# Supplementary material for: Central venous catheter–associated complications in pediatric patients diagnosed with Hodgkin lymphoma: implications for catheter choice
Source: Support Care Cancer. 2022 Jul 1;30(10):8069–79. doi: 10.1007/s00520-022-07256-3 (PMC9512752; doi:10.1007/s00520-022-07256-3)
Supplement: Supplementary file 3 — Supplementary file3 (DOCX 19 KB) [file 520_2022_7256_MOESM3_ESM.docx]

Central venous catheter associated complications in pediatric patients diagnosed with Hodgkin lymphoma: implications for catheter choice

**Journal title:** Supportive Care in Cancer

**Authors:** Ceder H. van den Bosch^1^, Judith Spijkerman^1^, Marc H.W.A. Wijnen^1^, Idske C.L. Kremer Hovinga^2^, Friederike A.G. Meyer-Wentrup ^1^, Alida F.W. van der Steeg^1^, Marianne D. van de Wetering^1^, Marta Fiocco^1,3,4^, Indra E. Morsing^1^, Auke Beishuizen^1^.

**Author affiliations:**

^1^ Princess Máxima Center for Pediatric Oncology, Utrecht, The Netherlands.

^2^ Van Creveldkliniek University Medical Centre Utrecht, Benign Hematology, Thrombosis and Hemostasis, Utrecht, The Netherlands.

^3^ Mathematical Institute, Leiden, The Netherlands

^4^ Leiden University Medical Center, Leiden, The Netherlands.

**Details corresponding author:**

C.H. van den Bosch, M.D. / PhD-student

C.H.vandenBosch-4@prinsesmaximacentrum.nl

ORCHID ID: 0000-0003-0612-578X

ONLINE RESOURCE 3 Case descriptions of CVT events

|  | Clinical characteristics thrombosis | | | | Patient related risk factors | | | | | | | | | |  | CVC related risk factors | | |
| --- | --- | --- | --- | --- | --- | --- | --- | --- | --- | --- | --- | --- | --- | --- | --- | --- | --- | --- |
| Pt | Severity thrombosis; complications | Diagnosis due to^a^ | Treatment | Days until CVT | A  g  e | Ann-Arbor; Mediastinal mass | GA not preferred | Sex | FH | OAC | Smoking | Obesity | Vein compression | Thrombophilia^b^ | Signs of infection during CVT | CVC type; Insertion side | CVC to vein ratio | TPN |
| 1 | subclavian | routine US | AC, removal | 84 | 16 | IV; + | - | M | - | - | - | - | - | ? | + | PICC SL; R | 0.33 | - |
| 2 | brachial/basilic-subclavian | symptoms | AC | 9 | 16 | III; + | - | F | - | + | ? | - | - | ? | - | PICC SL; R | 0.27 | - |
| 3 | jugular-brachiocephalic; VCSS, chylothorax | symptoms | AC, PTA, removal | 374 | 16 | II; + | - | F | - | + | + | + >30 | - | ? | + | TIVAP SL; R | ? | - |
| 4 | subclavian-atrium; PE2x, recurrent thrombosis atrium | symptoms | AC, TB, removal | 74 | 17 | III; + | - | F | - | - | - | + >30 | - | APS | - | PICC SL; R | 0.24 | - |
| 5 | brachiocephalic | symptoms | AC | 3 | 14 | II; + | + | F | - | - | ? | - | + VCS | ? | - | PICC SL; R | 0.18 | - |
| 6 | basilic-subclavian; CLABSI | symptoms | AC, removal | 13 | 17 | III; + | - | F | ? | + | ? | - | - | Heterozygote FV Leiden | + | PICC SL; R | 0.17 | - |
| 7 | basilic-jugular/brachiocephalic | symptoms | AC, removal | 7 | 17 | IV; + | - | M | + | - | ? | - | - | Negative | - | PICC DL; L | 0.25 | - |
| 8 | brachial-subclavian | symptoms | AC | 24 | 15 | II; + | - | F | ? | - | ? | - | + VCS | ? | + | PICC SL; R | 0.28 | - |
| 9 | basilic-brachiocephalic; post-thrombotic syndrome (Modified Villalta score 4) | symptoms | AC, removal | 10 | 13 | II; + | - | F | ? | - | - | - | - | ? | - | PICC SL; R | 0.29 | - |
| 10 | TIVAP tip 2-3cm | routine MRI | AC | 64 | 18 | III; + | - | F | ? | + | Passive | - | - | ? | - | TIVAP SL; L | ? | + |

HL; Hodgkin Lymphoma, Pt; Patient, US; Ultrasound, MRI; Magnetic Resonance Imaging, AC; Anticoagulants, TB; Thrombectomy, PE; Pulmonary Embolism, PTA; Percutaneous transluminal angioplasty, CLABSI; Central Line Associated Bloodstream Infection, CVT; Central Venous Thrombosis, M; Male, F; Female, FH; Family History, OAC; Oral Anti-conceptives, VCS; Vena Cava Superior, VCSS; Vena Cava Superior Syndrome, PICU; Pediatric Intensive Care Unit, GA; General Anesthesia, FV; Factor V, APS; Antiphospholipid Syndrome, SL; Single Lumen, DL; Double lumen, R; Right, L; Left, CVC; Central Venous Catheter, TIVAP; Totally Implantable Venous Access Port, PICC; Peripherally Inserted Central Catheter, TPN; Total Parenteral Nutrition, +; yes, -; no, ?; missing.

Risk factors that were similar for all ten patients: All patients were diagnosed with classic HL with a mediastinal mass, all CVCs were inserted with one insertion attempt, none of the patients received anticoagulants before the occurrence of the CVT, and none of the patients were admitted to the PICU before the thrombosis occurred.

^a^ Symptoms observed: swelling, redness, pain, and inability to flush or aspirate the CVC.

^b^ Tested due to recurrence of thrombosis (Pt 4), unexplained clinical symptoms of inflammatory disease (Pt 6) and positive medical family history (Pt 7).
