# Supplementary material for: Central venous catheter–associated complications in pediatric patients diagnosed with Hodgkin lymphoma: implications for catheter choice
Source: Support Care Cancer. 2022 Jul 1;30(10):8069–79. doi: 10.1007/s00520-022-07256-3 (PMC9512752; doi:10.1007/s00520-022-07256-3)
Supplement: Supplementary file 4 — Supplementary file4 (DOCX 26 KB) [file 520_2022_7256_MOESM4_ESM.docx]

Central venous catheter associated complications in pediatric patients diagnosed with Hodgkin lymphoma: implications for catheter choice

**Journal title:** Supportive Care in Cancer

**Authors:** Ceder H. van den Bosch^1^, Judith Spijkerman^1^, Marc H.W.A. Wijnen^1^, Idske C.L. Kremer Hovinga^2^, Friederike A.G. Meyer-Wentrup ^1^, Alida F.W. van der Steeg^1^, Marianne D. van de Wetering^1^, Marta Fiocco^1,3,4^, Indra E. Morsing^1^, Auke Beishuizen^1^.

**Author affiliations:**

^1^ Princess Máxima Center for Pediatric Oncology, Utrecht, The Netherlands.

^2^ Van Creveldkliniek University Medical Centre Utrecht, Benign Hematology, Thrombosis and Hemostasis, Utrecht, The Netherlands.

^3^ Mathematical Institute, Leiden, The Netherlands

^4^ Leiden University Medical Center, Leiden, The Netherlands.

**Details corresponding author:**

C.H. van den Bosch, M.D. / PhD-student

C.H.vandenBosch-4@prinsesmaximacentrum.nl

ORCHID ID: 0000-0003-0612-578X

ONLINE RESOURCE 4 Risk factor analysis for CVC-related CVT in pediatric Hodgkin lymphoma patients

|  |  |  | Patients without CVT events | | Patients with CVT events | | CVC-days | Incidence rate (IR) per 1 000 CVC-days | Comparison IRs or means |
| --- | --- | --- | --- | --- | --- | --- | --- | --- | --- |
|  |  |  | N | % | N | % | N |  | IRR (CI95%) |
| Patient related risk factors | Age at insertion | ≤13 years | 33 | 97.1 | 1 | 2.9 | 9725 | 0.10 | 1 |
|  |  | >13 years | 55 | 85.9 | 9 | 14.1 | 13283 | 0.68 | 6.59 (0.83-52.00) |
|  | Sex | Male | 48 | 96.0 | 2 | 4.0 | 11852 | 0.17 | 1 |
|  |  | Female | 40 | 83.3 | 8 | 16.7 | 11156 | 0.72 | 4.25 (0.90-20.01) |
|  | Hodgkin type | Classic | 84 | 89.4 | 10 | 10.6 | 22692 | 0.44 | 1 |
|  |  | NLPHL | 4 | 100.0 | 0 | 0.0 | 316 | 0.00 | Undefined |
|  | Mediastinal mass | No | 8 | 100.0 | 0 | 0.0 | 1255 | 0.00 | 1 |
|  |  | Yes | 80 | 88.9 | 10 | 11.1 | 21753 | 0.46 | Undefined |
|  | Ann-Arbor classification | ≤II | 41 | 91.1 | 4 | 8.9 | 7472 | 0.54 | 1 |
|  |  | >II | 47 | 88.7 | 6 | 11.3 | 15536 | 0.39 | 0.72 (0.20-2.56) |
|  | Thrombotic FH | Negative | 56 | 91.8 | 5 | 8.2 | 14036 | 0.36 | 1 |
|  |  | Positive | 4 | 80.0 | 1 | 20.0 | 1401 | 0.71 | 2.00 (0.23-17.15) |
|  | Anti-conceptives | No | 77 | 92.8 | 6 | 7.2 | 18319 | 0.33 | 1 |
|  |  | Yes | 11 | 73.3 | 4 | 26.7 | 4689 | 0.85 | 2.61 (0.74-9.23) |
|  | Anti-conceptive type | Progesterone | 3 | 75.0 | 1 | 25.0 | 3360 | 0.30 | 1 |
|  |  | Progesterone + estrogen | 9 | 75.0 | 3 | 25.0 | 1998 | 1.50 | 5.05 (0.52-48.49) |
|  | Smoking | No | 57 | 95.0 | 3 | 5.0 | 14015 | 0.21 | 1 |
|  |  | Yes | 2 | 66.7 | 1 | 33.3 | 1139 | 0.88 | 4.10 (0.43-39.43) |
|  |  | Passive smoking | 5 | 83.3 | 1 | 16.7 | 1774 | 0.56 | 2.63 (0.27-25.31) |
|  | Obesity at diagnosis | No | 72 | 90.0 | 8 | 10.0 | 17554 | 0.46 | 1 |
|  |  | Yes | 16 | 88.9 | 2 | 11.1 | 5454 | 0.37 | 0.81 (0.17-3.79) |
|  | Compression veins | No | 72 | 90.0 | 8 | 10.0 | 19502 | 0.41 | 1 |
|  |  | Yes | 16 | 88.9 | 2 | 11.1 | 3506 | 0.57 | 1.39 (0.30-6.55) |
|  | VCS compression | No | 76 | 90.5 | 8 | 9.5 | 20597 | 0.39 | 1 |
|  |  | Yes | 12 | 85.7 | 2 | 14.3 | 2411 | 0.83 | 2.14 (0.45-10.06) |
|  |  | <50% | 9 | 90.0 | 1 | 10.0 | 1624 | 0.61 | 1.59 (0.20-12.67) |
|  |  | >50% | 3 | 75.0 | 1 | 25.0 | 787 | 1.27 | 3.27 (0.41-26.15) |
|  | Thrombosis before insertion | No | 87 | 89.7 | 10 | 10.3 | 23008 | 0.43 | 1 |
|  |  | Yes | 1 | 100.0 | 0 | 0.0 | 0 | 0.00 | Undefined |
|  | Anticoagulant use at insertion | No | 86 | 89.6 | 10 | 10.4 | 22846 | 0.44 | 1 |
|  |  | Yes | 2 | 100.0 | 0 | 0.0 | 162 | 0.00 | Undefined |
|  | Thrombophilia | No | 1 | 50.0 | 1 | 50.0 | 321 | 3.12 | 1 |
|  |  | Yes | 1 | 33.3 | 2 | 66.7 | 475 | 4.21 | 1.35 (0.12-14.90) |
|  | Insertion with GA not preferred | No | 84 | 90.3 | 9 | 9.7 | 22333 | 0.40 | 1 |
|  |  | Yes | 4 | 80.0 | 1 | 20.0 | 675 | 1.48 | 3.68 (0.47-29.01) |
|  | PICU admission in history | No | 85 | 89.5 | 10 | 10.5 | 22652 | 0.44 | 1 |
|  |  | Yes | 3 | 100.0 | 0 | 0.0 | 356 | 0.00 | Undefined |
| CVC related risk factors | Long-term anticoagulant use during CVC-insertion | No | 85 | 89.5 | 10 | 10.5 | 19061 | 0.52 | 1 |
|  |  | Yes | 3 | 100.0 | 0 | 0.0 | 280 | 0.00 | Undefined |
|  | Complicated vein puncture | No | 82 | 89.1 | 10 | 10.9 | 18654 | 0.54 | 1 |
|  |  | Yes | 2 | 100.0 | 0 | 0.0 | 211 | 0.00 | Undefined |
|  | CVC type | No-PICC | 30 | 93.8 | 2 | 6.3 | 12263 | 0.16 | 1 |
|  |  | PICC | 58 | 87.9 | 8 | 12.1 | 7078 | 1.13 | 6.93 (1.47-32.63)* |
|  | CVC type | TIVAP | 29 | 93.5 | 2 | 6.5 | 12258 | 0.16 | 1 |
|  |  | Single lumen PICC | 50 | 87.7 | 7 | 12.3 | 6151 | 1.14 | 6.98 (1.45-33.57)* |
|  | CVC lumen number | Single | 79 | 89.8 | 9 | 10.2 | 18409 | 0.49 | 1 |
|  |  | >Single | 9 | 90.0 | 1 | 10.0 | 932 | 1.07 | 2.20 (0.28-17.32) |
|  | CVC side | Right | 78 | 90.7 | 8 | 9.3 | 17803 | 0.45 | 1 |
|  |  | Left | 10 | 83.3 | 2 | 16.7 | 1538 | 1.30 | 2.89 (0.61-13.63) |
|  | CVC use for TPN | No | 85 | 90.4 | 9 | 9.6 | 17780 | 0.51 | 1 |
|  |  | Yes | 3 | 75.0 | 1 | 25.0 | 1561 | 0.64 | 1.27 (0.16-9.99) |
|  |  |  | Mean | SD | Mean | SD |  |  | (CI95%) |
|  | CVC to vein ratio |  | 0.27 | 0.05 | 0.25 | 0.06 |  |  | (-0.02-0.06) |

CVC; Central Venous Catheters, CVT; Central Venous Thrombosis, TIVAP; Totally Implantable Venous Access Port, FH; Family History, NLPHL; Nodular lymphocyte-predominant Hodgkin lymphoma, TPN; Total Parenteral Nutrition, PICC; Peripherally Inserted Central Catheter, IR;
