# Supplementary material for: Central venous catheter–associated complications in pediatric patients diagnosed with Hodgkin lymphoma: implications for catheter choice
Source: Support Care Cancer. 2022 Jul 1;30(10):8069–79. doi: 10.1007/s00520-022-07256-3 (PMC9512752; doi:10.1007/s00520-022-07256-3)
Supplement: Supplementary file 5 — Supplementary file5 (DOCX 21 KB) [file 520_2022_7256_MOESM5_ESM.docx]

Central venous catheter associated complications in pediatric patients diagnosed with Hodgkin lymphoma: implications for catheter choice

**Journal title:** Supportive Care in Cancer

**Authors:** Ceder H. van den Bosch^1^, Judith Spijkerman^1^, Marc H.W.A. Wijnen^1^, Idske C.L. Kremer Hovinga^2^, Friederike A.G. Meyer-Wentrup ^1^, Alida F.W. van der Steeg^1^, Marianne D. van de Wetering^1^, Marta Fiocco^1,3,4^, Indra E. Morsing^1^, Auke Beishuizen^1^.

**Author affiliations:**

^1^ Princess Máxima Center for Pediatric Oncology, Utrecht, The Netherlands.

^2^ Van Creveldkliniek University Medical Centre Utrecht, Benign Hematology, Thrombosis and Hemostasis, Utrecht, The Netherlands.

^3^ Mathematical Institute, Leiden, The Netherlands

^4^ Leiden University Medical Center, Leiden, The Netherlands.

**Details corresponding author:**

C.H. van den Bosch, M.D. / PhD-student

C.H.vandenBosch-4@prinsesmaximacentrum.nl

ORCHID ID: 0000-0003-0612-578X

ONLINE RESOURCE 5 Risk factor analysis for CVC-related CVT in pediatric Hodgkin lymphoma patients only including SL PICC and TIVAP (N=88)

|  |  |  | Incidence rate (IR) per 1 000 CVC-days | Comparison IRs or means |
| --- | --- | --- | --- | --- |
|  |  |  |  | IRR (CI95%) |
| Patient related risk factors | Age at insertion | ≤13 years | 0.10 | 1 |
|  |  | >13 years | 0.66 | 6.27 (0.78-50.11) |
|  | Sex | Male | 0.09 | 1 |
|  |  | Female | 0.76 | 8.46 (1.06-67.64)* |
|  | Ann-Arbor classification | ≤II | 0.59 | 1 |
|  |  | >II | 0.33 | 0.57 (0.15-2.11) |
|  | Thrombotic family history | Negative | 0.38 | 1 |
|  |  | Positive | 0.00 | Undefined |
|  | Anti-conceptives | No | 0.29 | 1 |
|  |  | Yes | 0.87 | 2.98 (0.80-11.11) |
|  | Anti-conceptive type | Progesterone | 0.30 | 1 |
|  |  | Progesterone + estrogen | 1.57 | 5.29 (0.55-50.81) |
|  | Smoking | No | 0.22 | 1 |
|  |  | Yes | 0.88 | 3.92 (0.41-37.65) |
|  |  | Passive smoking | 0.64 | 2.88 (0.30-27.65) |
|  | Obesity at diagnosis | No | 0.42 | 1 |
|  |  | Yes | 0.40 | 0.96 (0.20-4.63) |
|  | Compression veins | No | 0.37 | 1 |
|  |  | Yes | 0.64 | 1.71 (0.35-8.21) |
|  | VCS compression | No | 0.36 | 1 |
|  |  | Yes | 0.93 | 2.60 (0.54-12.49) |
|  |  | <50% | 0.73 | 2.04 (0.25-16.60) |
|  |  | >50% | 1.27 | 3.56 (0.44-28.90) |
|  | Thrombophilia | No | 0.00 | 1 |
|  |  | Yes | 4.21 | Undefined |
|  | Insertion with GA not preferred | No | 0.38 | 1 |
|  |  | Yes | 1.48 | 3.90 (0.49-31.19) |
| CVC related risk factors | CVC type | TIVAP | 0.16 | 1 |
|  |  | Single lumen PICC | 1.14 | 6.98 (1.45-33.57)* |
|  | CVC side | Right | 0.47 | 1 |
|  |  | Left | 0.65 | 1.38 (0.17-11.04) |
|  | CVC use for TPN | No | 0.47 | 1 |
|  |  | Yes | 0.64 | 1.35 (0.17-10.79) |
|  | CVC to vein ratio | NA | NA | (-0.03-0.04) |

CVC; Central Venous Catheter, CVT; Central Venous Thrombosis, TIVAP; Totally Implantable Venous Access Port, GA; General Anesthesia, TPN; Total Parenteral Nutrition, PICC; Peripherally Inserted Central Catheter, IR; Incidence Rate, IRR; Incidence Rate Ratio, VCS; Vena Cava Superior, SD; Standard Deviation, CI; Confidence Interval. *Significant values
