# Supplementary material for: Central venous catheter–associated complications in pediatric patients diagnosed with Hodgkin lymphoma: implications for catheter choice
Source: Support Care Cancer. 2022 Jul 1;30(10):8069–79. doi: 10.1007/s00520-022-07256-3 (PMC9512752; doi:10.1007/s00520-022-07256-3)
Supplement: Supplementary file 6 — Supplementary file6 (DOCX 20 KB) [file 520_2022_7256_MOESM6_ESM.docx]

Central venous catheter associated complications in pediatric patients diagnosed with Hodgkin lymphoma: implications for catheter choice

**Journal title:** Supportive Care in Cancer

**Authors:** Ceder H. van den Bosch^1^, Judith Spijkerman^1^, Marc H.W.A. Wijnen^1^, Idske C.L. Kremer Hovinga^2^, Friederike A.G. Meyer-Wentrup ^1^, Alida F.W. van der Steeg^1^, Marianne D. van de Wetering^1^, Marta Fiocco^1,3,4^, Indra E. Morsing^1^, Auke Beishuizen^1^.

**Author affiliations:**

^1^ Princess Máxima Center for Pediatric Oncology, Utrecht, The Netherlands.

^2^ Van Creveldkliniek University Medical Centre Utrecht, Benign Hematology, Thrombosis and Hemostasis, Utrecht, The Netherlands.

^3^ Mathematical Institute, Leiden, The Netherlands

^4^ Leiden University Medical Center, Leiden, The Netherlands.

**Details corresponding author:**

C.H. van den Bosch, M.D. / PhD-student

C.H.vandenBosch-4@prinsesmaximacentrum.nl

ORCHID ID: 0000-0003-0612-578X

ONLINE RESOURCE 6 Risk factor analysis for CVC-related CVT in pediatric Hodgkin lymphoma patients only including SL PICC and TIVAP and excluding patients where general anesthesia was not preferred (N=83)

|  |  |  | Incidence rate (IR) per 1 000 CVC-days | Comparison IRs or means |
| --- | --- | --- | --- | --- |
|  |  |  |  | IRR (CI95%) |
| Patient related risk factors | Age at insertion | ≤13 years | 0.11 | 1 |
|  |  | >13 years | 0.59 | 5.42 (0.67-44.01) |
|  | Sex | Male | 0.09 | 1 |
|  |  | Female | 0.69 | 7.54 (0.93-61.31) |
|  | Ann-Arbor classification | ≤II | 0.49 | 1 |
|  |  | >II | 0.33 | 0.68 (0.16-2.85) |
|  | Anti-conceptives | No | 0.24 | 1 |
|  |  | Yes | 0.87 | 3.58 (0.90-14.32) |
|  | Anti-conceptive type | Progesterone | 0.30 | 1 |
|  |  | Progesterone + estrogen | 1.57 | 5.29 (0.55-50.81) |
|  | Smoking | No | 0.23 | 1 |
|  |  | Yes | 0.88 | 3.76 (0.39-36.17) |
|  |  | Passive smoking | 0.64 | 2.76 (0.29-26.56) |
|  | Obesity at diagnosis | No | 0.37 | 1 |
|  |  | Yes | 0.40 | 1.08 (0.22-5.34) |
|  | Compression veins | No | 0.36 | 1 |
|  |  | Yes | 0.56 | 1.03 (0.13-8.40) |
|  | VCS compression | No | 0.36 | 1 |
|  |  | Yes | 0.57 | 1.57 (0.19-12.75) |
|  |  | <50% | 0.73 | 2.01 (0.25-16.37) |
|  |  | >50% | 0.00 | Undefined |
| CVC related risk factors | CVC type | TIVAP | 0.16 | 1 |
|  |  | Single lumen PICC | 1.07 | 6.59 (1.33-32.63)* |
|  | CVC side | Right | 0.43 | 1 |
|  |  | Left | 0.65 | 1.53 (0.19-12.40) |
|  | CVC use for TPN | No | 0.43 | 1 |
|  |  | Yes | 0.64 | 1.49 (0.18-12.11) |
|  | CVC to vein ratio, mean (SD) | NA | NA | (-0.05-0.03) |

CVC; Central Venous Catheter, CVT; Central Venous Thrombosis, TIVAP; Totally Implantable Venous Access Port, TPN; Total Parenteral Nutrition, PICC; Peripherally Inserted Central Catheter, IR; Incidence Rate, IRR; Incidence Rate Ratio, VCS; Vena Cava Superior, SD; Standard Deviation, CI; Confidence Interval. *Significant values
